# Phyto-Extract-Mediated Synthesis of Silver Nanoparticles Using Aqueous Extract of *Sanvitalia procumbens*, and Characterization, Optimization and Photocatalytic Degradation of Azo Dyes Orange G and Direct Blue-15

**DOI:** 10.3390/molecules26206144

**Published:** 2021-10-12

**Authors:** Madeeha Aslam, Fozia Fozia, Anadil Gul, Ijaz Ahmad, Riaz Ullah, Ahmed Bari, Ramzi A. Mothana, Hidayat Hussain

**Affiliations:** 1 Department of Chemistry, Kohat University of Science & Technology, Kohat 26000, Pakistan; madeehaaslam06@gmail.com; 2 Biochemistry Department, KMU Institute of Medical Sciences, Kohat 26000, Pakistan; drfoziazeb@yahoo.com; 3 Beijing Key Laboratory for Green Catalysis and Separation, Department of Chemistry and Chemical Engineering, Beijing University of Technology, Beijing 100124, China; anadilgul97@gmail.com; 4 Department of Pharmacognosy, College of Pharmacy, King Saud University, P.O. Box 2457, Riyadh 11451, Saudi Arabia; rmothana@ksu.edu.sa; 5 Department of Pharmaceutical Chemistry, College of Pharmacy, King Saud University, P.O. Box 2457, Riyadh 11451, Saudi Arabia; abari@ksu.edu.sa; 6 Department of Bioorganic Chemistry, Leibniz Institute of Plant Biochemistry, Weinberg 3, D-06120 Halle, Germany; Hidayat.Hussain@ipb-halle.de

**Keywords:** green synthesis, *Sanvitalia procumbens*, silver nanoparticles, azo dyes degradation

## Abstract

Green synthesis of silver nanoparticles (AgNPs) employing an aqueous plant extract has emerged as a viable eco-friendly method. The aim of the study was to synthesize AgNPs by using plant extract of *Sanvitalia procumbens* (creeping zinnia) in which the phytochemicals present in plant extract act as a stabilizing and reducing agent. For the stability of the synthesized AgNPs, different parameters like AgNO_3_ concentration, volume ratios of AgNO_3_, temperature, pH, and contact time were studied. Further, AgNPs were characterized by UV–visible spectroscopy, FT-IR (Fourier Transform Infrared Spectroscopy), XRD (X-ray Diffraction), SEM (Scanning Electron Microscopy), and EDX (Energy Dispersive X-ray Spectrometer) analysis. FT-IR analysis showed that the plant extract contained essential functional groups like O–H stretching of carboxylic acid, N–H stretching of secondary amides, and C–N stretching of aromatic amines, and C–O indicates the vibration of alcohol, ester, and carboxylic acid that facilitated in the green synthesis of AgNPs. The crystalline nature of synthesized AgNPs was confirmed by XRD, while the elemental composition of AgNPs was detected by energy dispersive X-ray analysis (EDX). SEM studies showed the mean particle diameter of silver nanoparticles. The synthesized AgNPs were used for photocatalytic degradation of Orange G and Direct blue-15 (OG and DB-15), which were analyzed by UV-visible spectroscopy. Maximum degradation percentage of OG and DB-15 azo dyes was observed, without any significant silver leaching, thereby signifying notable photocatalytic properties of AgNPs.

## 1. Introduction

Metal nanoparticles have a variety of features in various fields. Since the sizes, dimensions, and compositions of metallic nanomaterials are all tied to their optical, physical and chemical properties, nanoscale materials have already been used in a wide range of applications [1]. Nanostructured metallic nanoparticles with extraordinary tunable complex surface plasmon, optoelectronics, biomedical, and catalytic properties were created owing to their high surface area to volume ratio and limited effort to the surface functionalization [2]. Despite the fact that many metals occur in nature, only elements such as silver, palladium, platinum and gold are widely synthesized for their nanostructured aspect [3]. Silver nanoparticles (AgNPs), among the metals listed above, have drawn a great deal of interest because of their unique properties for use in the textile industries, agriculture, water detoxification, pharmaceutics, and as a catalyst in oxidation reactions and air filtration [4,5,6,7]. Chemical, physical, electrochemical, irradiative, photochemical, and biological methods are now available for the synthesis of silver nanoparticles [8,9]. Since most of these methods involve the use of hazardous chemicals and extreme reaction conditions, this often results in chemical toxicity and environmental contamination. The detrimental effects of chemical processes, as well as the growing focus on green chemistry, have increased the importance of biological methods for the synthesis of AgNPs [10,11]. Because of their versatility, organic design, and profitability, biomediated methods are considered a viable alternative to physical and chemical methods. Several groups have reported on the synthesis of silver nanoparticles by using different plants extract like *Andrographis paniculata* [12], *Cathranthus roseus* [13] and *Rhynchosia suaveolens* [14], which act as a reducing and stabilizing agent.

Recently, silver nanoparticles (AgNPs) have risen in prominence due to their wide range of applications in biomedicine such as anticancer [15], antibacterial [16], and antioxidant activity [17]. AgNPs toxicity has been linked to an increase in the synthesis of reactive oxygen species (ROS), which causes the release of silver ions to occur [18]. ROS development may induce oxidative stress, which is basically the reported mechanism of AgNP’s toxicity, by lowering superoxide dismutase (SOD) and glutathione (GSH) levels and raising the lipid peroxidation levels in cells [19,20]. Nowadays, textile and synthetic dyes cause pollution that threaten living organisms and also contribute to changes in the climate. One of the possible strategies for achieving meaningful remediation is the catalytic degradation of synthetic pollutants by nanoparticles. Plant-mediated nanoparticles have proven to be effective photocatalytic agents in the fight against organic pollutants [21]. Numerous techniques like oxidation, adsorption, ion-exchange, chemical coagulation, and biological photocatalysis are available to overcome this issue [22]. AgNPs from *Phaseolus vulgaris* have recently been shown to degrade 4-nitrophenol, reactive red 141 dyes [23], and other fabricated AgNPs designed from *Solanum surattense,* which are utilized as an efficient catalyst towards Rhodamine B (RhB) [24]. Green synthesis of AgNPs has a wide range of applications for dye removal in a practical operating system that produces less sludges than other methods.

In the light of other discrepancies, different biological methods have reported on the synthesis of AgNPs and on the photocatalytic activities of other dyes. So, this work was carried out to synthesize AgNPs using *Sanvitalia procumbens* (*S. procumbens*) aqueous plant extract, and then to explore the photocatalytic applications of the synthesized AgNPs. The synthesis of AgNPs using aqueous extract of this plant and its photocatalytic degradation of azo dyes (Orange G and Direct blue-15) has not been reported in literature, and in this article we are reporting it for the first time. According to an ethnobotanical survey of weeds in the state of Mexico, *S. procumbens* (ornamental perpetually flowering plant) is one of the major plant families of commercial importance for medicinal, herbal, culinary, and ornamental uses [25]. Thus, the medicinally essential *S. procumbens* aqueous plant extract has since been used as a possible bioreducing agent for the green synthesis of AgNPs in such a cost-effective and environmentally sustainable manner. In light of the foregoing, the current study was conducted to produce low-cost, clear, and easily reusable AgNPs from *S. procumbens* that contains bioreductants and stabilizers such as alkamides, phenolics, proteins, and terpenoids, which are responsible for the reduction of Ag^+^ to Ag^0^ [25,26,27,28]. *Sanvitalia procumbens*-based silver nanoparticles can be highly reproducible. Large-scale utilization assessing the reproducible process can show the natural variability that occurs in the chemical composition of plant species that grow at the month of December, as well as assess how a plant can be used for this purpose so that the entire plant extract can be gainfully utilized in the process. Furthermore, the elemental composition, geometry, morphological, and optical study of synthesized AgNPs was carried out by using various techniques such as Energy Dispersive X-ray analysis (EDX), X-ray Diffractometer, Scanning Electron Microscope (SEM), Fourier Transform Infrared Spectroscopy (FT-IR), and UV–visible spectrophotometry. Moreover, the current study also assessed potential applications in the environment via green-synthesized AgNPs for the photocatalytic degradation of hazardous Orange G and Direct Blue-15 azo dyes by considering these dyes to be biodegradable.

## 2. Results and Discussion

Several studies have stressed and illustrated the possible use of shade-dried plants as a reducing and stabilizing source for nanoparticle synthesis. After integrating an aqueous plant extract of *S. procumbens* with the AgNO_3_ solution at a ratio of 1:9 (0.01 mol/L solution of AgNO_3_), the color change indicated that the synthesis of AgNPs occurred, where the plant extract was used as a reducing and stabilizing agent in this analysis. According to the literature, the initial indication of AgNPs was observed as a color transition from pale yellow to dark yellow and eventually colloidal brown, within a few minutes. The observed results were consistent with previous reports in which AgNPs were synthesized using various plant extracts, and aqueous silver nitrate solution (10^−3^ mol/L) altered the color from transparent to brown after extract addition [29,30].

### 2.1. UV-Visible Spectroscopy

**Figure 1:**
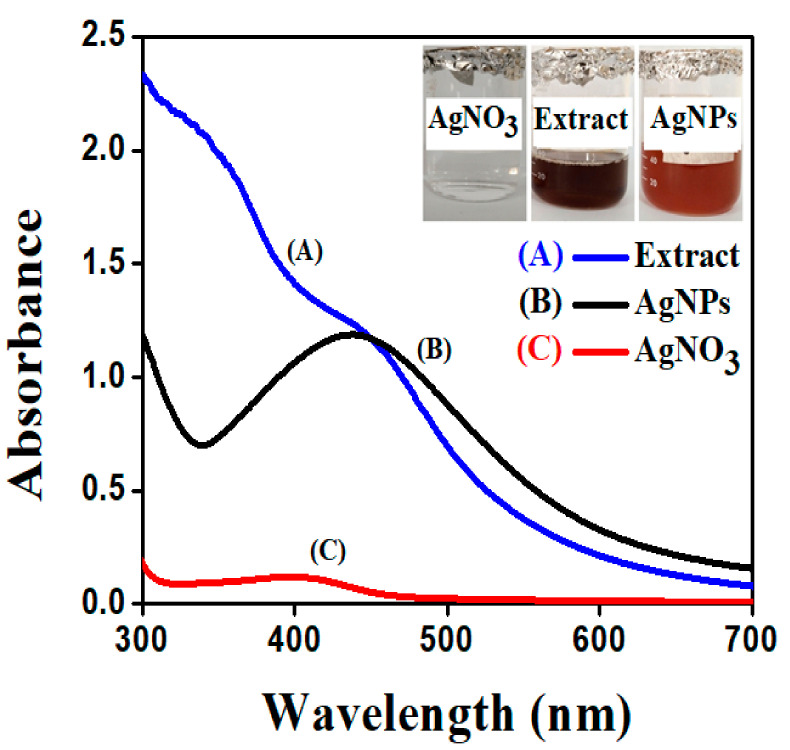
UV-Visible spectra of: (**A**) Pure extract of *S. procumbens*; (**B**) Synthesized AgNPs; and (**C**) 0.01 mol/L solution of AgNO_3_.

On the nanoscale level, one of the most intriguing features of metal nanoparticles is their optical properties, which vary proportionally with their size and shape. The large-size AgNPs are obtained at the weak absorbance peak and shift towards higher wavelength, while maximum absorbance peak shifts towards a lower wavelength when the particle size becomes smaller. In the current study, the absence of an absorbance peak in the visible region was examined when the plant extract and AgNO_3_ solution was observed in a UV-visible spectrophotometer. However, the synthesis of AgNPs was confirmed by a mixture of AgNO_3_ and plant extract. The AgNPs formation in aqueous colloidal solution showed the sharp SPR (surface plasmon resonance) peak recorded at 438 nm as shown in Figure 1. This is because plant extracts contain plenty of phytochemicals that aid in the reduction of AgNPs and serve as a capping agent [31]. Due to the coupled oscillation of electrons in light wave resonance of metal nanoparticles, particularly AgNPs, a free electron to absorb the surface plasmon resonance phenomenon is provided [32]. The achieved results are comparable to those reported in the literature, wherein the *Datura stramonium* plant extract was used to reduce silver nanoparticles, and UV-visible absorbance spectra revealed that the SPR (surface plasmon resonance) band for silver occurred in the 400–450 nm range. The reproducibility of AgNPs synthesis was confirmed using the aqueous extract of a previously collected batch of plant, which provided silver nanoparticles when the optimized ratio of plant extract and AgNO_3_ (1:9 mL) was used [33,34].

#### 2.1.1. Effect of AgNO_3_ Concentration

**Figure 2:**
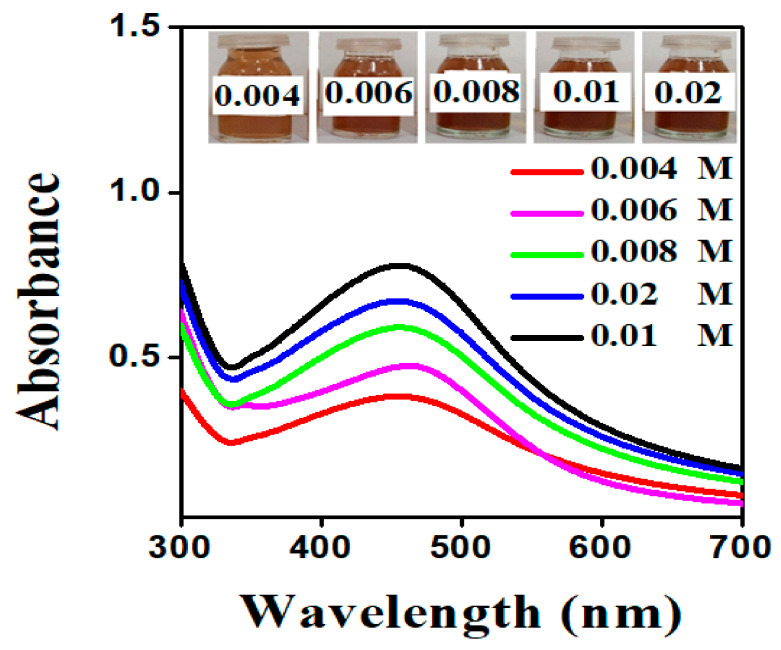
UV-visible spectrum of synthesized AgNPs from *S. procumbens* aqueous extract at various concentrations of AgNO_3_.

This parameter was measured by varying AgNO_3_ concentrations under ideal circumstances. The size and forms of AgNPs were affected by varying the concentration of AgNO_3_ solution. In this analysis, SPR (surface plasmon resonance) peak showed that at lower AgNO_3_ concentrations, AgNPs with larger size distributions were present, implying the presence of AgNPs with broader size distributions. The SPR peak became sharper and narrower as the concentration of AgNO_3_ rose, along with an increase in its intensity [35]. This suggests that AgNPs with a narrow particle size distribution were involved when the concentration of AgNO_3_ solution was increased. However, if the concentration of AgNO_3_ further increases to 0.02 mol/L, the intensity of such SPR peak weakens and broadens due to agglomerations induced by Van der Waals interactions between adjacent nanoparticles. It was concluded that with 0.01 mol/L AgNO_3_ solution, a distinct peak was found at 438 nm, but as stated in the literature, the concentration rises from 0.01 mol/L, and the intensity peak of synthesized nanoparticles decrease, which was also mentioned in Figure 2 [36].

#### 2.1.2. Effect of AgNO_3_ Volume to Plant Extract Ratios

**Figure 3:**
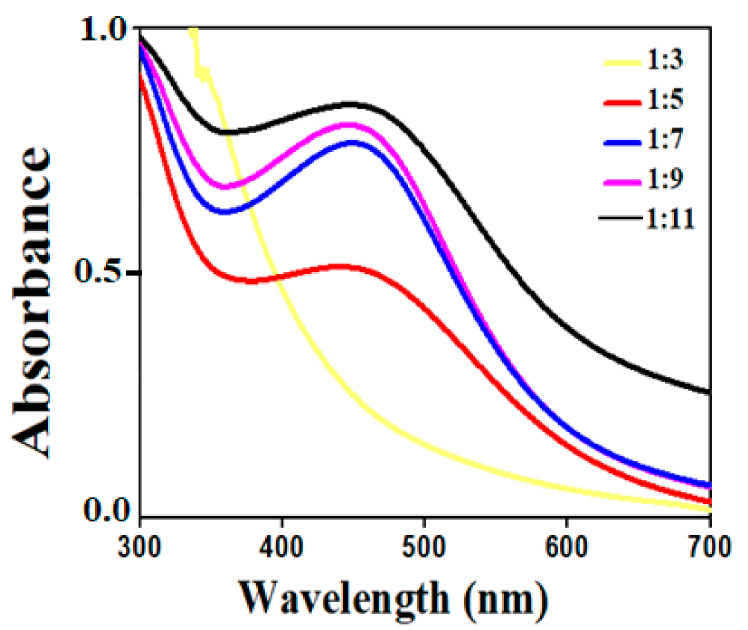
Effect of AgNO_3_ volume to *S. procumbens* plant extract ratios.

To decide the impact of volume ratios on AgNPs, different extracts and AgNO_3_*v*/*v* ratios were used. The transition in spectrum was observed at each ratio, as seen in Figure 3, where intensity increases as the ratios rises from 1:3 to 1:9; the peak becomes sharp as volume increases. The size and shape of AgNPs are also effected by increasing the volume of AgNO_3_ solution, as mentioned in Figure 3, which showed that, with increasing the volume of AgNO_3_ solution, the peak intensity also increased, indicating that small-size AgNPs were obtained. While at lower volume of AgNO_3_ solution, non-uniform large-sized AgNPs were synthesized [37]. As a result, from the given data, the maximum absorbance spectra were recorded at 438 nm for 1:9 *v*/*v* ratio, beyond this the peak becomes broader. The absorbance showed the quantity of AgNPs formed due to the reduction of silver ions.

#### 2.1.3. Effect of Temperature on AgNPs

**Figure 4:**
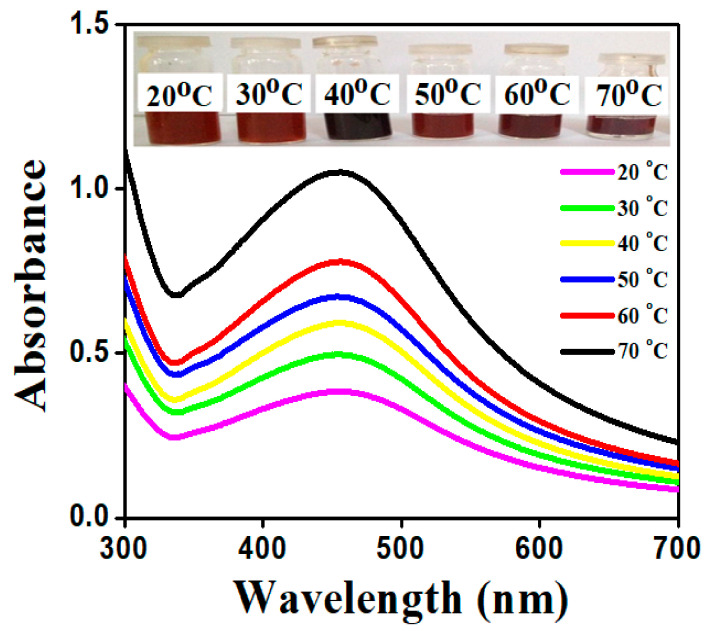
Absorption spectra of AgNPs achieved at different reaction temperatures using *S. procumbens* plant extract AgNPs.

The production of AgNPs is heavily influenced by temperature. The reaction mixture of synthesized AgNPs was placed at various temperatures from 20–70 °C for 30 min. The color and intensity shift of the reaction mixture is illustrated in Figure 4. In general, silver nanoparticles aggregate together easily. The color changes from brown to black as shown in Figure 4, which is due to more aggregation within the particles at 40 °C. The structure and size of the particles formed during the aggregation were too large and lost the property of surface plasmon resonance. Hence, the synthesized AgNPs were stable up to 70 °C, but the more intense absorbance peak observed at 70 °C was due to the formation of small size AgNPs. This also implies that, at high temperature, the kinetic energy of the molecules increases and silver ions gets consumed faster, leaving less possibility for particle size growth. Thus, smaller particles of nearly uniform size distribution are formed at higher temperature. It was discovered that absorbance increased with increasing temperature [38]. Hence, the maximum intensity peak of 438 nm at 70 °C resulted.

#### 2.1.4. Effect of Time on AgNPs

**Figure 5:**
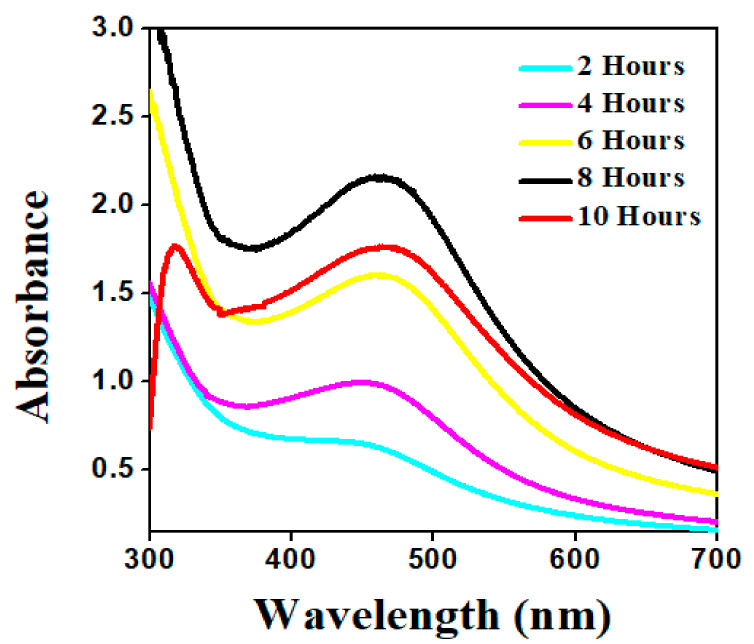
Absorption spectra of AgNPs obtained at different time intervals.

The impact of reaction time was studied by regularly reviewing the reaction mixture of an aqueous plant extract and AgNO_3_ solution at different time intervals. The AgNO_3_ solution reacts with the plant extract, resulting in a color shift within 1 h of the reaction. With the aging process, the color became more intense [39]. According to UV–visible spectrophotometric measurements taken at regular intervals of time, the broad surface plasmon resonance peak was obtained between 0 h and 2 h, which was due to the sluggish conversion of silver ion (Ag^+^ to Ag^0^). Since a significant amount of Ag^+^ was transformed to Ag^0^, an outstanding SPR (surface plasmon resonance) spectrum was found as the reaction time increased. The absorption peak of synthesized AgNPs was observed in the UV–vis spectra with strong SPR in the range of 438 nm at 8 h, indicating fast synthesis (Figure 5) [40].

#### 2.1.5. Effect of pH on AgNPs

**Figure 6:**
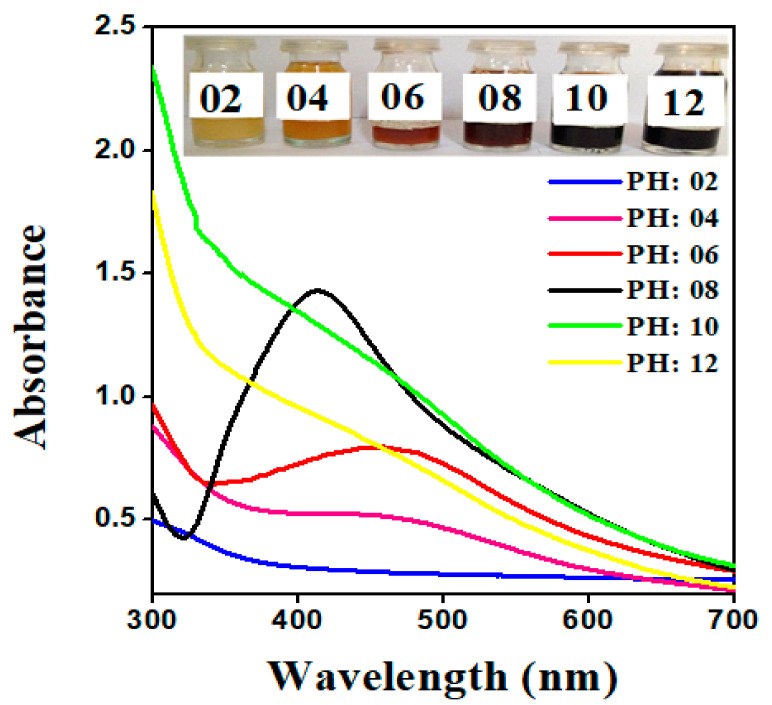
Normalized absorption spectra of AgNPs at different pH values of the reaction mixture employed S. *procumbens* plant extract.

The potential of the reaction pH is to comprehend the electrical charges of biomolecules, which may affect their reducing and capping capabilities, as well as the subsequent growth of the nanoparticles. The impact of pH on the absorption spectra of synthesized AgNPs via plant extract was shown in Figure 6. AgNPs were configured at various pH levels: 2, 4, 6, 8, 10, and 12. The absorption peak shifted towards lower wavelength, implying that the size of synthesized AgNPs was shrinking with the increasing pH of the reaction mixture. Furthermore, it was also noticed that the rate of reduction and the color of the solution turned colloidal brown faster at higher pH. As a result, the more favorable synthesis of AgNPs occurs at alkaline pH; this could be accredited to the ionization of the functional groups at higher pH. The slow rate of reduction observed in the acidic medium could be attributed to the electrostatic repulsion of anions present in the reaction mixture. Referring to the previously reported literature, the SPR (surface plasmon resonance) peak of AgNPs was not observed in the range of 400 to 500 nm at pH 2, 4, and 6 [41]. Whereas in the current study, the sharp and narrow SPR (surface plasmon resonance) peak was obtained within the range of 438 nm at pH 8, as shown in Figure 6, inferring that OH groups were responsible for a reduction of Ag^+^ as supported by Davidovic et al. [42].

#### 2.1.6. Stability of AgNPs

The stability test of synthesized AgNPs was checked at 5, 10, and 15 days after synthesis. According to the results, there are no vibrational shifts at the surface plasmon resonance of AgNPs. The presence of a sharp peak indicated the development of mono-dispersed nanoparticles. As reported, from an aqueous extract of *Cassia roxburghii,* synthesized AgNPs were found to be stable for 2 months in a previous analysis [43].

### 2.2. Fourier Transform Infrared Spectroscopy (FT-IR) Analysis

**Figure 7:**
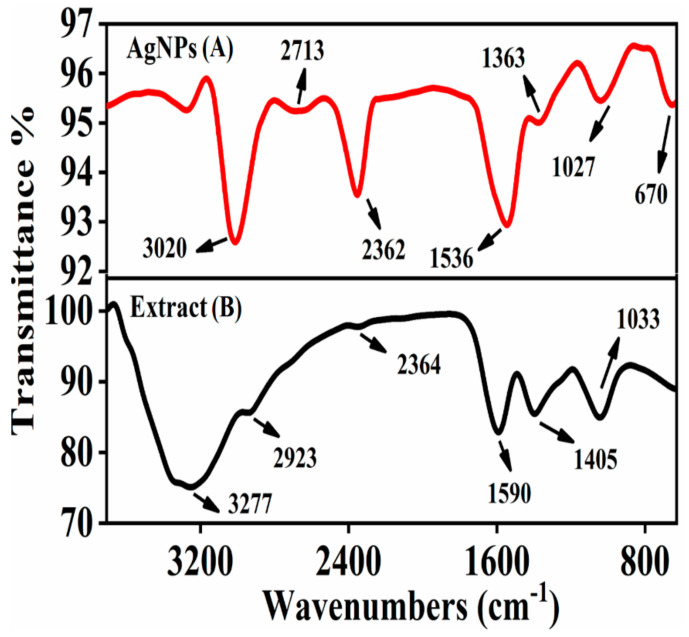
FTIR spectra of synthesized AgNPs (**A**) and *S. procumbens* plant extract (**B**).

The synthesis of AgNPs was achieved by biologically active functional groups and interaction sites present in an aqueous plant extract of *S. procumbens* that acts as a reducing, capping, and stabilizing agent, which were identified by using FT-IR (Bruker, Alpha-II) spectroscopy. The data suggests that during AgNPs synthesis, the phytochemicals present in a plant extract play an important role in the production of metal nanoparticles, which reduce the metal salt into its nanoparticles. It is tough to select an individual phytochemical entity, but in general, flavonoids, phenolic groups, organic acids, and proteins all are thought to be essential for the synthesis of nanoparticles [44]. In FT-IR spectroscopy, *S. procumbens* extract showed prominent bands of absorbance at around 3277, 2923, 2364, 1590, 1405, and 1033 cm^−1^ (Figure 7)**.** The phenolic groups and proteins act as capping and stabilizing agents, which play an important role in the production of AgNPs; this was confirmed by a band at 3277 cm^−1^, which was responsible for O–H stretching vibration of carboxylic acid [45]. The small band at 2923 cm^−1^ was due to **C**–H stretching of alkane, whereas the band at 2364 cm^−1^ represents the C–O vibration [46,47]. The band at 1590 cm^−1^ showed the N–H stretching vibration of amines present in proteins and 1405 cm^−1^ aligns to the C–N stretching of aromatic amine groups [48]. The 1033 cm^−1^ band was assigned C–O stretching vibration of alcohols, ester, or carboxylic acid. The reduction in bands at 3020, 2713, 2362, 1536, 1363, 1027, and 670 cm^−1^ is attributed to biomolecules present in the plant extract, which reduced Ag^+^ ions to Ag^0^. We theorized that the synthesized AgNPs were capped by biomolecules that were involved in silver nanoparticles formation [49].

### 2.3. X-ray Diffraction Analysis (XRD)

**Figure 8:**
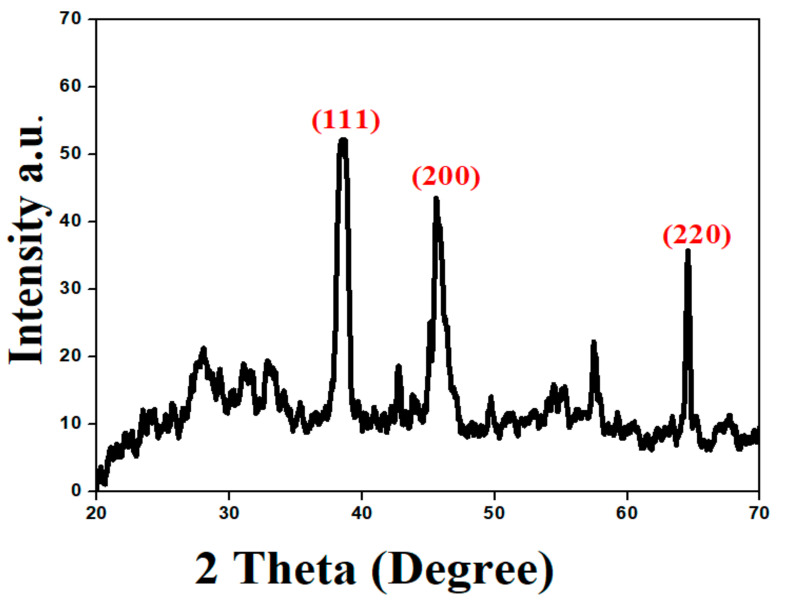
X-ray diffraction pattern of AgNPs synthesized from *S. procumbens*.

To investigate the crystalline nature and surface morphology of the synthesized AgNPs, the X-ray diffraction (XRD) analysis was performed in the range of 20–70 degrees at 2θ. The XRD pattern of the AgNPs synthesized by the reaction of silver salt solution with an aqueous plant extract of *S. procumbens* is shown in Figure 8. In XRD analysis, the crystal structure depends on the arrangement of atoms in a specific plane; the XRD peak showed higher intensity due to more atomic counts accumulated in its orientation. So, synthesized AgNPs, by using a plant extract of *S*. *procumbens*, were observed at high-intensity peaks at around 38°, 45°, and 64°. The mentioned peaks correspond to the *hkl* planes (111), (200), and (220) Bragg reflections, respectively, which were the exact peak positions as given for the face center cubic (FCC) lattice structure of AgNPs. This diffraction pattern was very similar to JCPDS file no. 04–0783 standard diffraction pattern [50]. The 38° peak was explained by assuming that the peak intensity of XRD becomes smaller along with the decrease in crystallite size. These facts revealed that the measuring content with the crystallites sizes of AgNPs were smaller than 30 nm [51]. The particle size of synthesized AgNPs was 26 nm, calculated by employing Scherrer’s equation.

(1) $$D=\frac{0.9\lambda }{\beta \cos\theta }$$

where *D* is crystallite size, *λ* corresponds to the X-rays wavelength that used 1.5406 °A, *β* is FWHM (Fill width half maximum), and *θ* corresponds to Bragg’s angle.

### 2.4. Energy Dispersive X-ray (EDX) Analysis

**Figure 9:**
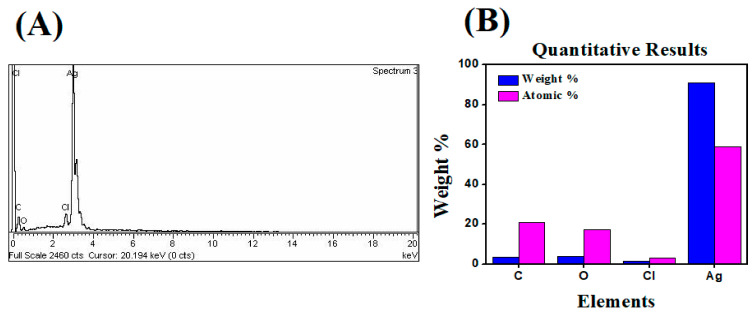
(**A**) Energy dispersive X-ray spectrum for AgNPs from *S. procumbens*; (**B**) Weight percentage of atoms present in AgNPs.

The elemental composition was determined by using energy dispersive X-ray (EDX) analysis. The EDX result of synthesized AgNPs revealed the existence of silver, as well as the residual carbon, oxygen, and chlorine peaks that were unwittingly inserted from the surface molecule of the plant extract. Green-synthesized AgNPs were responsible for the observed C, O, Cl, and Ag peaks. The peak of Ag at 3 eV confirms the formation of silver nanoparticles, where standard weight percent of silver was 90.88%. In this analysis, the green-synthesized AgNPs showed aggregated morphologies and a nonuniform size distribution (Figure 9). The particle size is dependent on how the aqueous extract is administered during the synthesis. Likewise, in accordance with these statements, the presence of Ag, Cl, and O were observed within the spectrum that appeared around 3 keV, which indicated the existence of elemental silver in the biosynthesized AgNPs [52].

### 2.5. Scanning Electron Microscopy (SEM)

**Figure 10:**
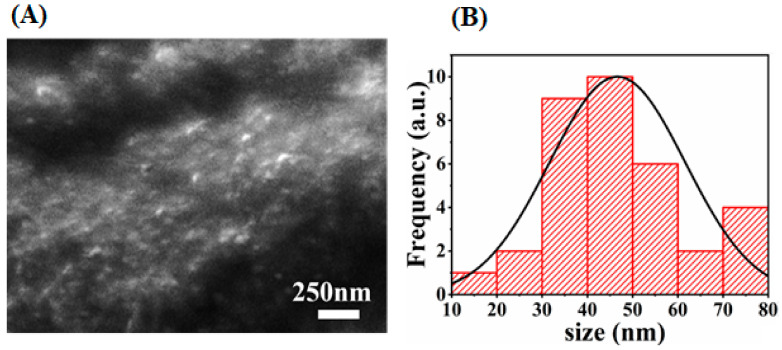
SEM image of AgNPs with 250 nm scale bar (**A**), and their corresponding size distribution histogram (**B**).

Researchers used scanning electron microscopy (SEM) to design the images of the sample and consider the morphology of the nanoparticles. The shape and size of synthesized AgNPs by using green synthesis were shown in a micrograph of a scanning electron microscope (SEM). According to the given micrograph, the scale of the synthesized AgNPs was up to 250 nm. The micrograph revealed that the particle sizes range from 10–80 nm and are spherical in shape. Most of them are synchronized in the form of large clusters, but few of them are dispersed as seen in Figure 10. So, the average particle size was found to be 46 nm, which is consistent with XRD results of 26 nm (crystallite size).

### 2.6. Photocatalytic Degradation Analysis

#### 2.6.1. Photocatalytic Degradation of Orange G Azo Dye

**Figure 11:**
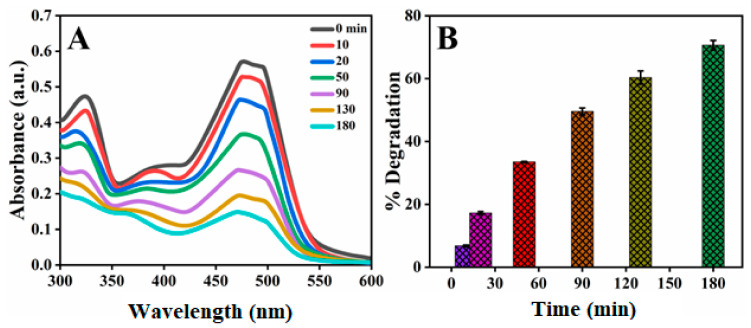
(**A**) Absorbance spectra of Orange G (15 ppm) dye at different time intervals, (**B**) Percentage degradation of Orange G dye at different time intervals.

To investigate the photocatalytic capability of stable AgNPs towards the degradation of various organic dye molecules, the commonly used Orange G dye in many dyeing industries was selected. Orange G is a well-known water-soluble dye that has always had widespread usage in industries such as leather, textiles, pharmaceuticals, paper, and printing [53]. To evaluate dye degradation in the presence of AgNPs as a catalyst, a UV–vis spectrophotometer was used. The high absorption peak of OG was centered around 483 nm; the reaction of degradation was carried out at room temperature. The photocatalytic degradation of dye was monitored by changing the absorbance peak. Degradation of OG did not proceed in the absence of synthesized AgNPs. Experiments were performed by using 0.05 g/100 mL of synthesized AgNPs, and the results were expressed in terms of dye degradation. Figure 11 showed the photocatalytic degradation of OG versus time. It was concluded that the maximum photocatalytic degradation of OG dye was ascertained by noting the time required for the OG absorption peak to reach baseline. Pertaining to this principle, the upper limit percentage of OG dye degradation was found to be 70.61% at about 180 min with *S. procumbens*-AgNPs [11].

#### 2.6.2. Photocatalytic Degradation of Direct Blue-15 Azo Dye

**Figure 12:**
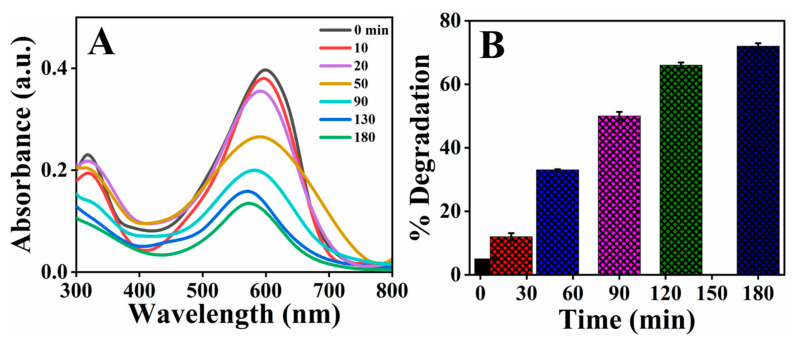
(**A**) Absorbance spectra of Direct Blue-15 (10 ppm) dye at different time intervals, (**B**) Percentage degradation of direct Blue-15 dye at different time intervals.

Dye pollution has grown in tandem with the development of industries that use colorants, such as in clothing, leather, food, and agrochemicals. Since the majority of commonly used azo dyes are made from benzidine, these dyes contain a carcinogenic substance that is hazardous to marine biota. The azo dye DB-15 used in the textile had a variety of toxic effects on microalgae, Cladocera, and zebrafish embryos. So, the dumping of this colorant waterbody should be controlled to prevent environmental impacts [54]. As a result, deterioration of the azo dye DB-15 in wastewater is strongly recommended to alleviate their harmful impacts. A UV–visible spectrophotometer was used to monitor the extent of degradation by green-synthesized AgNPs. The absorption maxima for BD-15 were recorded at a wavelength of 602 nm. However, in the presence of 0.05 g/100 mL AgNPs as a catalyst, the decrease in absorption peak of dye was observed as the time progressed, as shown in Figure 12. To determine the maximum photocatalytic degradation of DB-15, the amount of time required for the absorbance peak to reach baseline was measured. At 180 min, the maximum percentage of DB-15 dye degradation was found to be 70%. However, the synthesized catalyst showed more degradation efficiency against OG dye than DB-15, which might be due to the complex structure of DB-15 with many N atoms and its difficult conversion into oxidized nitrogen.

## 3. Materials and Methods

### 3.1. Plant Collection

The *S. procumbens* plant was harvested from the area of Kohat, Khyber Pakhtunkhwa, Pakistan and was identified by Dr. Nisar Ahmed, Department of Plants and Environmental Sciences, Kohat University of Science & Technology.

### 3.2. Preparation of Sanvitalia procumbens Aqueous Plant Extract

The plant of *S. procumbens* was washed thoroughly with tap water to remove debris and other contamination, followed by washing with distilled water. The plant was shade-dried at room temperature. Twenty grams of finely cut plant was heated at 70 °C for 30 min in 200 mL of distilled water. The extract was cooled down and then filtered via Whatman No. 1 filter paper, and the aqueous plant extract was stored in the refrigerator for further analysis.

### 3.3. Green Synthesis of AgNPs

At room temperature, 1 mL volume of aqueous plant extract was added to 9 mL of 0.01 mol/L AgNO_3_ solution in a dropwise manner. After mixing the aqueous plant extract into silver ion solution, the reaction mixture was placed onto a rotatory orbital shaker operating at 200 rpm, then it was subjected to a water bath for 70 °C. Complete reduction of Ag^+^ to Ag^0^ ions in the reaction mixture was confirmed by the color change of AgNO_3_; plant extract solution from pale yellow to dark brown implied the formation of AgNPs (further scanned by UV-visible spectrophotometry). Finally, the samples were centrifuged for 15 min at 6000 rpm. The organic moieties were removed after centrifugation, and the pellet was washed with distilled water and methanol. The powder form of nanoparticles was obtained, which were utilized for further characterization and photocatalytic studies.

### 3.4. Effects of Different Parameters on the Stability of AgNPs

Green synthesized AgNPs were produced by using *S. procumbens* aqueous plant extract and different parameters including AgNO_3_ concentration, volume, pH, time, and temperature were studied to check the stability of AgNPs. Spectrophotometric study of AgNPs after 6–8 h of incubation in the dark at room temperature revealed the optimal conditions for efficient reduction of silver ions and eventual nucleation into silver nanoparticles. The impact of plant extract volume on green synthesis of AgNPs was explored through executing the reaction with various volumes of AgNO_3_ solution (1, 3, 5, 7, 9, and 11 mL), whilst maintaining the volume of plant extract remains as constant. The reaction mixtures were deposited with various AgNO_3_ concentrations, which were used to investigate the impact of AgNO_3_ concentration on AgNPs synthesis (0.004, 0.006, 0.008, 0.01, and 0.02 mol/L). So, the optimal AgNO_3_ concentration measured the absorbance of nanoparticles by UV-visible spectrophotometer. The aforementioned reaction mixture was subjected at different temperature conditions, namely 20 °C, 30 °C, 40 °C, 50 °C, 60 °C, and 70 °C, for synthesizing AgNPs. Following the synthesis, every single reaction tube was held at the specified temperature for 15 min. The pH effect was observed by exposing a reaction mixture of AgNPs at various pH level, and 0.1 mol/L NaOH solution was used to change the pH of the reaction solution to the optimal level.

### 3.5. Characterization of AgNPs

To calculate the reduction of silver nitrate into silver nanoparticles, UV-visible spectroscopy was used on a regular basis (Shimadzu-UV-1800). The AgNPs sample was diluted with distilled water and then examined under UV-visible spectrophotometer. The quartz cuvette cell filled with distilled water was used as a reference for recording the spectrograph at scanning speed of 300–700 nm [55]. The morphology of AgNPs was investigated by using SEM (SIGMA HV-Carl Zeiss, Oberkochen, Germany); this the sample of AgNPs was put onto a carbon grid, and the remaining sample was washed with blotting paper. Synthesized AgNPs were subsequently allowed to dry for 15 min under a mercury lamp before being imaged. A high-pressure mercury lamp was used to excite the storage molecules or proteins that exhibited autofluorescence [56]. At a 10 kV accelerating voltage, the characterization procedure of SEM was carried out. XRD was used to assess the crystalline nature and average grain size of AgNPs (XPERT-PRO). CuKa radiation was used to monitor X-ray diffraction in the 2θ range from 20–70 at 40 kV/40 mA current with dried AgNPs on a XRD grid [57]. The production of AgNPs was responsible for biologically active compounds that act as a reducing, capping, and stabilizing agents, which were identified by using a KBr pellet system in FT-IR (Bruker, Alpha-II) analysis. FT-IR (Bruker, Alpha-II) (Billerica, MA, USA) was performed by using a KBr pellet method to identify the functional groups of biological active compounds present in plant extract that act as reducing and capping agents for the formation of AgNPs. This spectral analysis was performed at 4 cm^−1^ resolution, within the range of 4000 to 500 cm^−1^ [58,59].

### 3.6. Photocatalytic Degradation Assay

The photocatalytic degradation studies were performed by mixing 100 mL of the reaction mixture into a 250 mL glass beaker, distinctly. Prior to being irradiated, the reaction sample (0.05 g/100 mL) was mixed with (15 ppm) OG and (10 ppm) DB-15 azo dye solution, respectively. Both solutions were constantly stirred for 30 min in the dark to ensure that equilibrium was reached. An absorption capacity of pure AgNPs for OG was evaluated at room temperature in the dark condition. Then the solution was irradiated by a Hg lamp with a high-pressure (max = 254 nm, 60 W, located 10 cm above the sample) sealed in to a rectangular steel box. The reactors were put on a magnetic stirrer to ensure that the solvent was evenly dispersed. After the irradiation times, aliquots of a previously utilized sample were removed at specific time intervals. Finally, the solution was centrifuged for 10 min at 4000 rpm to extract the suspended particles and then examined with a UV–visible spectrophotometer. The experiment was repeated twice, and the standard deviation did not reach 5%, indicating that the findings were relevant. The percentage degradation was calculated by using the following equation on the base of standard curve y = x0.0386 − 0.035 (R^2^ = 0.9935) and y = x0.034 − 0.0428 (R^2^ = 9.997) for OG and DB dyes, respectively.

(2) $$\%\mathrm{Degradation}=100\times \left(A_{0}-A\right)/A_{0}$$

where, ‘A_0_’ is the initial absorbance of Orange G and DB-15 azo dyes, and ‘A’ shows the absorbance of dyes solution obtained at each time interval [60].

## 4. Conclusions

In the current study, the synthesis and optimization of AgNPs was demonstrated by using plant aqueous extract of *S. procumbens*. The synthesized AgNPs were successfully characterized by using different techniques like UV-Visible, FT-IR, SEM, XRD, and EDX. Furthermore, AgNPs exhibited potential photocatalytic degradation activity of Orange G and Direct blue-15; the synthesized catalyst showed more degradation efficiency against OG dye than DB-15. Overall, all these results suggest that AgNPs, as synthesized in this study, can be used as a potent photodegradation analyte.

## Data Availability

Provided all data in the MS.
